# Flight Stressors: Pathophysiological Principles Guiding Safe Fixed-Wing Aeromedical Transport of Critically Ill Patients

**DOI:** 10.7759/cureus.102770

**Published:** 2026-02-01

**Authors:** Anastasia Tasiou, Christos Tzerefos, Insa K Janssen, Maria D Karagianni, Konstantinos Peramatzis, Eleni Tsianaka, Nurperi Gazioğlu, Nese Keser, Maria Karampouga, Stiliana Mihaylova, Niina Salokorpi, Aysegul Esen Aydin, Mary Murphy

**Affiliations:** 1 Department of Neurosugery, University Hospital of Larisa, Larisa, GRC; 2 Department of Neurosurgery, Hôpitaux Universitaires de Genève, Geneva, CHE; 3 Department of Neurosugery, Larissa General University Hospital, Larissa, GRC; 4 Department of Aviation Engineering, School of Aviation, Australian University, Safat, KWT; 5 Department of Neurosurgery and Quality, Kuwait Hospital, Sabah Al Salem, KWT; 6 Department of Neurosurgery, Istinye University Faculty of Medicine, Istanbul, TUR; 7 Department of Neurosurgery, University of Health Sciences Fatih Sultan Mehmet Training and Research Hospital, Istanbul, TUR; 8 Department of Neurological Surgery, Center for Cranial Base Surgery, University of Pittsburgh Medical Center, Pittsburgh, USA; 9 Department of Neurological Surgery, Nicosia General Hospital, Nicosia, CYP; 10 Department of Neurosurgery, University Hospital Sv. Ivan Rilski, Sofia, BGR; 11 Department of Neurosurgery, Oulu University Hospital, Oulu, FIN; 12 Department of Neurosurgery, Research Unit of Clinical Neuroscience, Medical Research Center, Oulu University, Oulu, FIN; 13 Department of Neurosurgery, Bakirkoy Research and Training Hospital for Neurology, Neurosurgery and Psychiatry, Istanbul, TUR; 14 Department of Vascular Neurosurgery, National Hospital for Neurology and Neurosurgery, London, GBR

**Keywords:** aeromedical transfer, en route deterioration, in-flight stressors, medical advices, medics, pathophysiology of air travel

## Abstract

International travel increases the chance of patients requiring aeromedical evacuation due to unexpected medical emergencies or worsening chronic medical conditions. Although critically ill patients are exclusively managed by specialized teams, all medics manage patients who may require air transfer after stabilization and first-tier management. These patients may face unpredictable and potentially harmful conditions at high altitudes. The aim of this study is to highlight in-flight factors that may negatively affect patients' well-being en route, providing useful information for all medics. A comprehensive literature search was conducted on PubMed and Google Scholar, up to March 2025, employing a multimethod approach to identify all relevant studies for this review. The search continued until no new citations emerged. Basic principles of physics are applied to clinical situations, demonstrating the relationship among pressure, volume, and temperature, which might affect pathophysiological processes of the human body under certain circumstances. Unique flight stressors, including the hypobaric aeronautical environment, thermal stress, gravitational forces, vibration, and noise, may adversely affect patients’ health. In-flight hypoxia is the most important risk during air transfer. Other hazardous conditions include the tendency for increased risk of thromboembolic events, as well as the potential for gas embolism, gas expansion in normally pneumatized body cavities, and malfunction of pneumatically operated medical devices. Even small amounts of trapped gas can lead to serious complications. This narrative review provides valuable information regarding in-flight stressors and experiences that physicians must be aware of and predict for safe aeromedical evacuation of critically ill patients.

## Introduction and background

The aeromedical transport of critically ill patients has its early beginning in 1784, and its role has expanded significantly over the past century [1-3]. Aeromedical transfer is required when emergency ground transportation is impossible or unsafe due to difficult conditions and/or long distances. Aeronautical evacuation could also be an option for patients who need to be transferred in high-volume centers of care. Patients who travel abroad for work, vacation, or outdoor activities may sometimes need or choose to return home to continue their medical treatment. Likewise, patients living abroad may wish to receive their treatment in the country of residence (medical repatriation).

Several unique challenges could appear en route. The aeronautical environment is remote from equipment and any kind of assistance available on the ground. Fastidious communication and meticulous planning before transfer are extremely important [4,5]. Various adverse events, such as unforeseen weather changes, transport delays, or rapid patient decline, may complicate the process of air transfer. Logistic aspects may limit this process, while optimal timing of an air transfer is always critically important [5]. A number of parameters, known as flight stressors related to unexpected conditions in-flight, need special attention. Altitude, gravity, humidity, temperature, acceleration, noise, and vibration could all negatively affect the patient during a transfer. Undoubtedly, the benefit of air transfer should justify the risk of in-flight deterioration.

The purpose of this study is to highlight basic pathophysiological characteristics of aviation at high altitude and define medical conditions that could be critically affected en route. We also intend to provide basic knowledge for physicians who may be confronted with patients requiring aeromedical transfer, after primary stabilization.

## Review

Methods

This work was conceived as a narrative review intended to give clinicians a concise, concept-driven account of the pathophysiological issues related to “flight stressors” during aeromedical evacuation. To assemble the evidence, we searched PubMed and Google Scholar from database inception through March 2025, using combinations of the terms aeromedical transport, air evacuation, pathophysiology of air travel, decompression sickness, gas expansion, aerospace pressure effects, and hypoxia or hypoxemia. Only English language sources were considered. We included any data associated with at least one predefined stressor domain, namely, altitude and gas laws, thermal or humidity effects, gravity and acceleration, noise or vibration, thromboembolism, medical device performance, or the logistical isolation that characterizes in-flight care. We excluded helicopter studies, purely animal experiments, abstracts without full texts, and opinion pieces that offered no physiological or clinical data.

Two reviewers (AT and IKJ) independently screened titles and abstracts and read the full texts of any citation with uncertain eligibility. Disagreements were resolved through discussion. For each eligible item, we extracted publication type, population or scenario, the stressor(s) addressed, and the headline physiological or clinical finding. Study designs ranged from bench simulations to cohort series. We synthesized the material qualitatively rather than attempting a pooled statistical analysis. During analysis, the evidence was clustered into seven thematic blocks that map directly onto our manuscript: altitude-related physiology, thermal and humidity stress, gravity and acceleration forces, noise and vibration with space constraints, thromboembolic risk, medical device behavior, and operational isolation. The review was prepared in accordance with the SANRA (Scale for the Assessment of Narrative Review Articles) guidelines to ensure methodological rigor in literature search, synthesis, and reporting [6].

Results

The search returned 2,525 unique records. After duplicate removal and language screening, 122 full‑text articles were thoroughly reviewed. Eighty-one peer‑reviewed studies and guidelines or technical documents met the inclusion criteria of our study. The research base was heterogeneous, comprising a diverse range of evidence. Varied studies, such as guidelines, hypobaric-physiology/coagulation studies, operational and aeromedical reports, case reports/series on pneumocephalus or air embolism, and technical documents on pressurization and in-flight equipment, were included in our narrative review. Due to heterogeneity in design, population, and outcome, a meta-analysis was not feasible.

Discussion

Fundamental Principles of Physics

**Figure 1:**
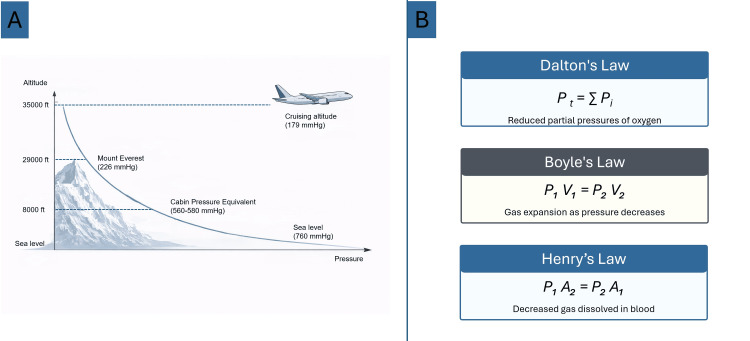
Relationship between altitude and ambient pressure and schematic illustration of fundamental physical laws governing gas behavior. Panel A demonstrates the decrease in ambient pressure with increasing altitude, from sea level to typical aircraft cabin altitude. Panel B summarizes Boyle’s, Dalton’s, and Henry’s laws, highlighting their relevance to physiological changes experienced by the human body during flight. Source: Ref. [4].

Understanding of the physical gas laws is essential for comprehending the specific stress experienced by the human body during flight. Breathing air consists of about 78% nitrogen, 21% oxygen, and 1% inert gases. At mean sea level, the pressure exerted by Earth’s atmosphere on a surface is conventionally set to 1013.25 hPa (hectopascals), equal to 760 mmHg. Basic physical principles describe the connection between pressure, volume, and temperature in a fixed quantity of gas [7]. According to these laws, although air composition remains constant at any level of altitude, the atmospheric barometric pressure decreases exponentially with increasing altitude [4]. In modern commercial aircraft, the 8,000-ft cabin pressure standard minimizes the physiological effect of rapid ambient pressure changes, allowing commercial aircraft to operate at cruising altitudes up to 45,000 ft (Figure 1) [4].

Dalton's Law

According to Dalton’s law, the overall pressure (P_t_) exerted by a gas mixture is determined by the additive contribution of the partial pressures (P_i_) of its constituent gases [7]. Although the fractional concentration of oxygen remains constant at increasing altitudes, the absolute oxygen partial pressure declines in parallel with the reduction in ambient atmospheric pressure (Figure 1) [7].

The atmospheric pressure at sea level is approximately 760 mmHg, depending on weather conditions. Oxygen at sea level consists of only 21% of the air, corresponding to 160 mmHg. As altitude increases, ambient atmospheric pressure progressively declines, resulting in a reduction of oxygen partial pressure from approximately 160 mmHg at sea level to about 80 mmHg [7]. This reduction directly limits the amount of oxygen available for respiration and underlies the development of hypoxia during flight [7].

Boyle's Law

Boyle’s law explains the inverse association between pressure (P) and volume (V) in a closed gas system, whereby a reduction in ambient pressure leads to proportional gas expansion, a principle fundamental to altitude-related physiological effects [8,9]. Within the hypobaric conditions of flight, gas contained in body cavities may increase in volume by as much as 30% [10]. This phenomenon is clinically relevant in situations involving non-communicating gas collections, such as pneumocephalus, pneumothorax, or intraluminal bowel gas in the setting of obstruction (Figure 1).

Henry's Law

Henry’s law describes the dependence of gas solubility (A) in liquids on the partial pressure (P) exerted by the gas above the fluid [4]. Under conditions of reduced ambient pressure at altitude, gas solubility decreases proportionally, allowing dissolved gases to come out of solution. This mechanism is classically illustrated by decompression sickness occurring during rapid ascent in divers with pulmonary barotrauma. Comparably, air embolism may serve as a model of clinical deterioration associated with flight-related hypobaric exposure (Figure 1).

Flight Stressors

One must consider unique factors, which we label flight stressors, that need special attention en route. These refer to conditions that may negatively affect the patient in-flight, including altitude, temperature, humidity, gravity, noise, vibration, limited space, and isolation.

Altitude

In-Flight Hypoxia

One of the most important risks in-flight due to the hypobaric aeronautical environment is hypoxia [4]. According to Dalton’s law, the partial pressure of oxygen decreases with increasing altitude. Although the effect of hypoxia varies, it is thought to begin at 10,000 ft altitude [7]. Most commercial flights reach altitudes between 25,000 and 45,000 ft, but modern pressurized cabins maintain cabin pressure at 8,000-ft altitude, ensuring the balance between passengers’ comfort and structural integrity [11,12].

A healthy individual inhales approximately 8 liters of air per minute at sea level; this value decreases by one-quarter at an altitude of 8,000 feet [13]. Healthy passengers easily compensate for this reduction by hyperventilating [8]. However, it may adversely affect ill patients who usually tolerate this reduction with a 2 L/min additional oxygen supply [14,15]. This phenomenon is even worse in patients with respiratory disorders. For patients already hypoxic at sea level, altitude has a profoundly negative effect on hemoglobin oxygen saturation. Patients with a resting oxygen saturation above 95% at sea level generally do not require supplemental oxygen during flight, whereas those with a saturation below 92% do require in-flight oxygen support [16]. In-flight hypoxia may also appear due to intestinal gas expansion, which may compress the diaphragm and further impair its function. This can be a significant problem in spinal-injured patients or those with intestinal obstruction.

Conditions such as heart failure, hematologic disease, renal impairment, and sleep apnea can heighten vulnerability to hypoxia related to altitude exposure [8,11]. Reduced tissue oxygenation is especially problematic in patients with traumatic brain injury, sinus infections, compartment syndromes of the extremities, ischemia at bowel anastomosis sites, or other gastrointestinal pathologies, as well as in burn injuries and soft-tissue wounds, where the risk of infection may be increased [17,18].

Altitude-related hypoxia can also trigger activation of the coagulation cascade [19,20]. Exposure to high altitude is associated with a transient prothrombotic state, mediated in part by hypoxia-induced release of plasminogen activator inhibitor-1 from vascular endothelial cells [21,22]. In addition, hypoxia modifies endothelial gene transcription, leading to a reduction in normal anticoagulant activity [23].

Hypoxia as a contributory factor to syncopal episodes on board is underrecognized [18]. It could also be responsible for dysrhythmias. Other important factors include pre-existing heart problems, dehydration, and nerve reflexes due to a sudden drop in cerebral blood flow [4,18]. Hypobaric hypoxia may further worsen the neurological injury in neurosurgical patients, promoting seizures and secondary brain and/or spinal ischemia [24,25].

Exposure to high altitude can be fatal if supplemental oxygen is not provided [7]. At altitudes up to 34,000 ft, delivery of 100% oxygen via a tight-fitting mask can approximate sea-level tissue oxygenation [7]. Beyond this, or in the absence of adequate oxygenation, a positive-pressure oxygen system is required until descent to a safe altitude [26]. Standard cabin pressurization combined with supplemental oxygen is essential to prevent hypoxia, which can be readily monitored using continuous pulse oximetry [8]. Avoiding smoking for at least 48 hours before a flight may also be beneficial [27].

Gas Embolism

The term includes two distinct entities: decompression sickness, which occurs due to bubble generation from inert gases previously dissolved in tissues, and arterial air embolism, in which air originating from the alveoli or venous system gains access to the arterial circulation through pulmonary vessels or intracardiac shunts [28,29]. Under hypobaric flight conditions, gas embolism develops as dissolved oxygen and nitrogen are liberated from the blood and surrounding tissues [8].

Decompression sickness: Bubble formation in the blood and tissues due to rapid pressure changes is responsible for the development of decompression sickness. Symptoms are mostly benign, including joint pain, rash, radiating abdominal pain, hypesthesia, paresthesia, dyspnea, malaise, fatigue, lightheadedness, and confusion [30]. Less common but more serious symptoms are motor weakness, ataxia, pulmonary edema, cardiopulmonary dysfunction, shock, and death [29,31]. Fatal outcomes are rare [30].

Reports in the literature relate departure control system (DCS) to flights during military operations [32]. The US Air Force reported 16 confirmed incidents of Central Nervous System DCS in 13 pilots (2002-2009). In most cases, symptoms were well-recognized and well-interpreted within 4 h. However, in some cases, symptoms recurred a few days later but responded well to hyperbaric oxygen therapy. Neuropsychiatric symptoms persisted in six pilots, representing permanent injury. All cases occurred in combat situations in association with the exposure to frequent altitude changes [32].

Harrison reported a knowledge gap that DCS has been studied in the military and not in general aviation (GA) [33]. The authors calculated the risk of DCS during flights in unpressurized piston GA aircraft capable of flying in high altitudes (>18,000 ft) and found that this risk was approximately 2%. However, no one needed medical attention [33].

No diagnostic workup is available to identify DCS [34]. Military flight crew, members, and trainees undergo special training in flight altitude chambers to be familiar with and capable of recognizing signs and symptoms of hypoxia or other altitude-associated physiological changes [35]. Severe symptoms usually appear within one to three hours of decompression, and 98% of the cases develop clinical symptoms within the first 24 hours [36,37]. Supplementary oxygen supply along with aggressive oral hydration is the first-line treatment, which is usually definitive for the majority of these patients [31]. In severe cases, a hyperbaric oxygen therapy in combination with intravenous hydration is highly recommended [32]. The clinical severity of the illness, the clinical response to treatment, and the presence of persistent or residual symptoms after the initial recompression define the optimal management and also the number of treatments required [37]. Prognosis is based on symptom severity, and avoidance of rapid altitude changes is crucial for preventing this syndrome.

Air embolism: Air embolism happens when air bubbles get into the bloodstream. If the air enters an artery, symptoms may include losing consciousness, confusion, neurological problems, irregular heartbeat, or signs of reduced blood flow to the heart. In contrast, when air enters a vein, it can cause low blood pressure, rapid breathing, low carbon dioxide levels, fluid in the lungs, or even cardiac arrest [38]. Surgical procedures that carry the highest risk of gas embolism, either venous or arterial, are sitting position craniotomy, cesarean section, hip replacement, and cardiac surgery with cardiopulmonary bypass [39]. Mechanical ventilation might also lead to arterial air embolism [40].

Cerebral air embolism during air travel is extremely rare [41]. Khawar et al. reported a case presenting with seizures and loss of consciousness on an international flight 20 minutes after takeoff [42]. Similarly, Edwardson et al. described another case who presented with a fatal stroke during air travel [41]. In both cases, the proposed mechanism was the expansion of a pre-existing pulmonary cyst with subsequent erosion of the adjacent pulmonary veins [41,42]. The deficit is usually reversible, and the treatment, if needed, includes 100% oxygen administration and hyperbaric therapy [43,44].

Gas Expansion

Body cavities: The hypobaric environment causes gas expansion in normally pneumatized body cavities, leading to pressure and discomfort in ears and sinuses (aerotidis, aerosinusitis, and aerodontalgia) [45]. This causes the familiar popping sound from the middle ear during takeoff [46]. Negative pressure in the middle ear creates nasal congestion in patients with middle ear infections, allergies, or sinusitis, acute or chronic, responsible for pain, tinnitus, vertigo, and/or hearing loss. Recent ear surgical procedures are a contraindication to flight [47].

Trapped gas may also lead to tympanic membrane damage, sinus rupture, surgical wounds dehiscence or rupture, expansion of pre-existing pneumothorax and/or pneumocephalus, gastrointestinal expansion, diaphragm distention associated with decreased functional capacity of the lungs, and abdominal barotrauma related to further respiratory compromise [4]. Even slight increases in the volume of trapped gas within confined compartments, such as the intracranial or intraocular spaces, may result in severe or catastrophic consequences.

Pneumothorax carries a substantial risk of recurrence, estimated at approximately 50% despite apparent clinical recovery, with the majority of relapses occurring within the first year and a lower incidence observed among individuals who have ceased smoking [48]. Guidance from the British Thoracic Society and the International Air Transport Association (IATA) recommends postponing air travel for a minimum of two weeks following radiographic confirmation of resolution in cases of traumatic pneumothorax [49,50]. More recent data indicate that flying as early as 72 hours after chest tube removal may be acceptable, if or when complete re-expansion of the lung has been achieved [51].

Healthy patients usually cope with the pressurized environment in a normal commercial flight, but this is not the rule for patients suffering head or spinal trauma [8]. These patients are not able to react with physiological compensation or express their pain due to an altered state of consciousness or intubation. Although aircraft cabin restrictions usually minimize barotrauma, traumatic or iatrogenic air pouches in vital structures can lead to life-threatening conditions [4].

Individuals who have recently undergone thoracic, abdominal, neurosurgical, or ophthalmologic procedures may be at risk of complications caused by altitude-related expansion of residual intrabody gas. Intestinal gas volume can increase by approximately 25% under hypobaric conditions. In the presence of postoperative ileus or bowel obstruction, such expansion may precipitate wound dehiscence, bleeding, and/or intestinal perforation [4]. Consequently, postponing air travel for at least 7-10 days following laparoscopic or other gas-insufflation surgical interventions is considered appropriate [47].

Pneumocephalus:* *The most well-documented deterioration upon air transfer is pneumocephalus [52]. Pneumocephalus, which could be postoperative following neurosurgical or ENT (ear, nose, and throat) surgeries, posttraumatic, or cranio-basal bone defects from infection or tumor, is considered an absolute contraindication to air travel [53-55]. In-flight expansion of the intracranial air results in tension pneumocephalus, leading to brain compression, increased intracranial pressure (ICP), decreased cerebral perfusion pressure and oxygenation, and subsequent neurological deterioration and/or death [18,52,56,57]. Lim et al. recommended a threshold of 20 ml of intracranial air for safe air travel [56].

The permissibility of air travel in patients with pneumocephalus remains controversial and continues to be debated in the literature [56]. Currently, no consensus exists regarding the appropriate timing for patients to fly following cranial surgery, particularly when pneumocephalus is present [56]. For cases involving emergency craniotomy before repatriation, it is advisable to postpone aeromedical transport for a minimum of 14 days postoperatively or until complete resorption of the pneumocephalus has occurred [53]. The IATA stipulates that patients who have undergone cranial surgery may only travel on commercial flights if they are free of intracranial air and are in satisfactory general health [49]. It is also common practice to perform a head CT scan to confirm the absence of intracranial air before proceeding with air transfer [58].

In a systematic review, Bichsel et al. found numerous reports of patients with pneumocephalus, including tension pneumocephalus, that have been safely transported in commercial aircraft and air ambulances without ICP increase and/or permanent neurological deficit [59]. Similarly, Donovan et al. reported that no patient with pneumocephalus (0.6-42.7 ml) sustained temporary or permanent neurologic decline due to air transportation [52]. Despite the need for additional clinical data to more accurately evaluate the safety of aeromedical transport in this patient population [60], an active postoperative cerebrospinal fluid leak constitutes an absolute contraindication to air travel. Bichsel et al. reported that ongoing extracranial-intracranial fistulous communication represents the sole identified risk factor associated with in-flight complications related to pneumocephalus [59].

Equipment: Medical equipment incorporating air-filled components, including urinary catheters, enteral feeding tubes, intravenous fluid bags, pneumatic splints, and other closed infusion devices, may be affected by gas expansion under hypobaric conditions. Endotracheal tube cuffs require particular attention. During ascent, partial cuff deflation is advised to avoid excessive tracheal wall pressure, which may cause mucosal injury, cuff rupture, or vagally mediated bradycardia [4]. In contrast, cuff underinflation during descent predisposes patients to aspiration pneumonia [4]. Maintaining stable cuff pressure throughout flight may be achieved by filling the cuff with normal saline [4] or by employing an automated device designed to regulate endotracheal tube cuff pressure [61].

Intravenous fluid bags should be used with infusion pumps because gas expansion can lead to disconnection or an increased air pocket in the tube/lines. Pneumatic splints are not allowed by many airlines. Bivalve casts can prevent circulatory problems [47].

Temperature, Humidity, and Gravity

As altitude increases, both temperature and humidity decrease. Thermal stress is a serious issue at high altitude. The cooling effect described by the standard lapse rate typically corresponds to a 2°C decrease in ambient air temperature for every 1,000 ft of elevation gain [10]. To mitigate this, cabin temperature is usually regulated within a comfortable range of approximately 18°C-30°C [62]. With modern pressurized aircraft, temperature remains stable, protecting the travelers from hazardous temperature shifts [45]. Temperature control is important, especially for patients who face difficulties in regulating their core temperature, e.g., thermodysregulation in spinal cord injury patients [63]. Due to low humidity, patients may experience thickened secretions, dehydration, increased mucous plugs, and thromboembolic events [46,64]. The thickened mucous plugs predispose patients with any respiratory difficulty to developing a lower respiratory tract infection in the week after travel.

In critically ill individuals, physiological responses to gravitational forces are amplified [64]. These patients may develop arterial hypertension, cardiac arrhythmias, redistribution of intravascular and interstitial fluids, tachycardia, elevated IC, reduced cerebral tissue oxygenation, and diminished venous return with consequent reductions in cardiac output. [64]. Changes in acceleration forces and vibrations possibly exacerbate the negative impact on the ICP and the postinjury spinal stability. Traumatic brain injury patients can experience noticeable increases in ICP and fluctuations in cerebral perfusion during takeoff or landing, which might be associated with a significant risk of serious deterioration and death [10,58]. Patients with high-level spinal cord injury tend to become very hypotensive during takeoff because blood drains to their feet. Spinal shock leads to neurogenic hypotension due to vasodilation and bradycardia, particularly during the early spinal shock phase. Volume expansion is usually ineffective, and patients often require vasopressors [65]. It is recommended to use norepinephrine over ephedrine due to α-adrenergic efficacy in autonomic dysfunction [66].

Acceleration, in conjunction with factors such as vibration, excessive noise, fluctuations in barometric pressure, and changes in partial oxygen pressure, may adversely affect both the ischemic core and the surrounding penumbra in stroke patients [24]. Additionally, acceleration stress can influence the accuracy of non-invasive blood pressure measurements, potentially resulting in erroneous readings. Management strategies for decreased humidity and altered gravitational forces include appropriate fluid administration and optimal patient positioning, head forward on takeoff, and head aft on landing in supine position [41].

Thromboembolic Phenomena

Long flights are associated with an increased risk of venous thromboembolic phenomena; the first case was reported in 1946 [67]. Most cases involve deep vein thrombosis (DVT) in the lower limbs followed by pulmonary embolism (PE). Also, there are reports of cerebral venous and arterial thrombosis [68-71].

Multiple mechanisms contribute to the elevated risk of venous thromboembolism (VTE) associated with air travel, including venous stasis, hypoxemia, and dehydration [72]. Individual susceptibility to flight-related VTE is influenced by factors such as age over 40 years, female sex, oral contraceptive use, lower-extremity varicosities, obesity, and inherited thrombophilic conditions [46]. In addition, many intracranial neoplasms are associated with a hypercoagulable state, and the postoperative period following craniotomy further increases thrombotic risk. Limb edema in affected extremities may also augment the likelihood of thrombus formation. Notably, the majority of passengers who experience DVT or PE during or after air travel have pre-existing risk factors, including prior thromboembolic events, recent hospitalization, surgery, and trauma - particularly spinal injury, malignancy, smoking, pregnancy, or exposure to estrogen-based therapies [72,73]. Prolonged flights, especially those exceeding eight hours, appear to confer an additional increase in risk [74].

Preventive strategies include minimizing prolonged immobility; encouraging ambulation when feasible, performing flexion, extension, and rotation of the ankles to promote circulation in the lower limbs; and ensuring adequate hydration during flight [46,72]. In travelers at higher risk, prophylactic use of low-molecular-weight heparin and graduated compression stockings is strongly recommended [53,75,76].

Noise, Vibration, and Limited Space

**Figure 2:**
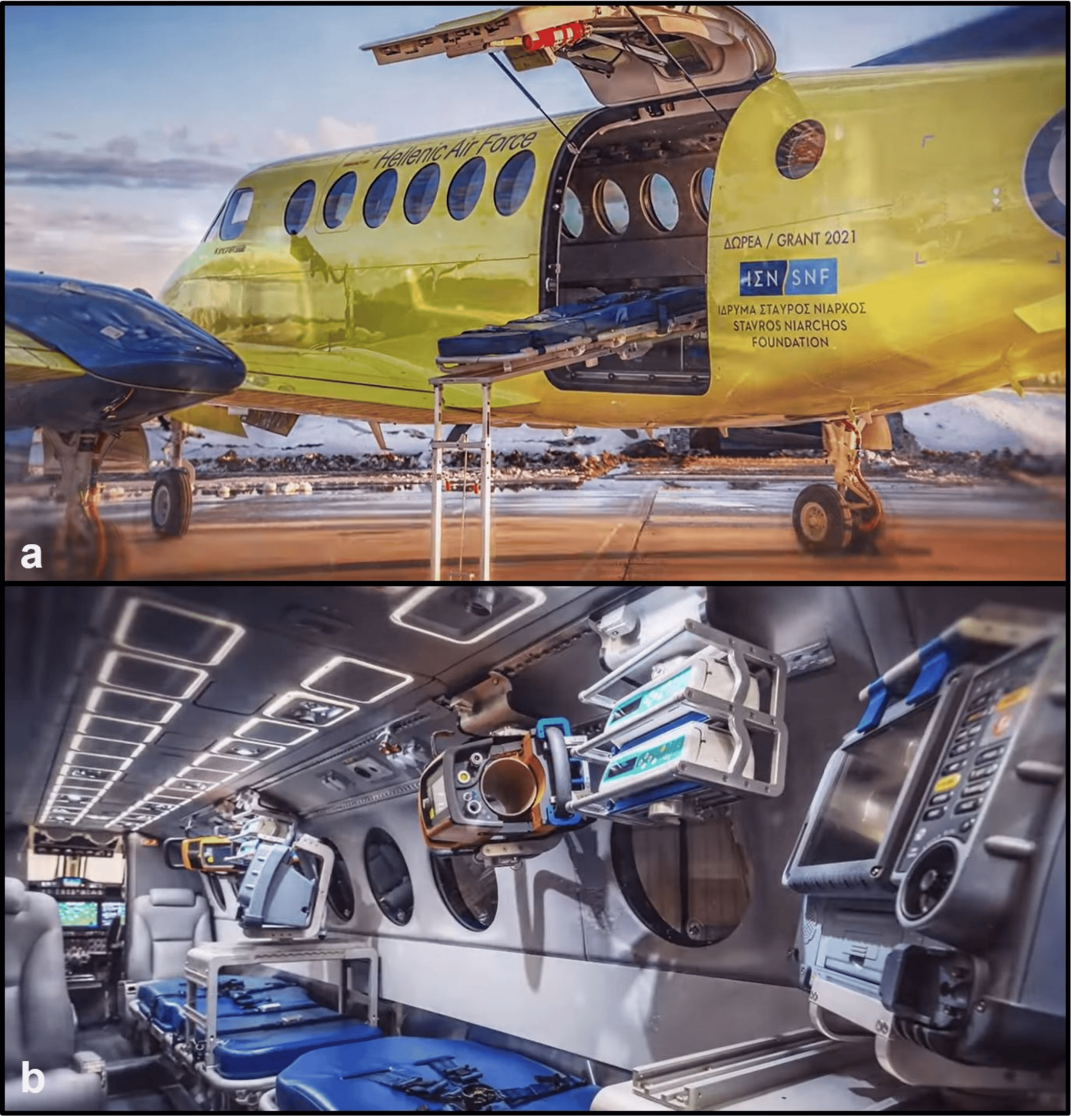
Aircraft Beechcraft King Air 350: (a) outside view and (b) internal configuration for aeromedical transportation Copyright: Hellenic Air Force/Press office. Published with permission.

Air ambulance aircraft are generally small with fixed wings and a lot of turbulence during the flight (Figure 2) [8]. Vibration levels are much higher in rotor-wing than in fixed-wing flights [77]. These factors should be considered in the interpretation of pulse oximeters and cardiac monitor measurements. Inserting venous lines is also difficult due to vibration. Making several stops during a lengthy trip can expose patients to acceleration, deceleration, turbulence, and vibrations, all of which may negatively impact their health.

The aviation setting is characterized by high ambient noise levels, which render auscultation with a stethoscope largely impractical and significantly hinder effective communication among the pilot, medical personnel, and patient. Excessive noise may also interfere with accurate clinical assessment. Combined exposure to noise, vibration, and spatial constraints increases physical discomfort, pain, nausea, and anxiety; may precipitate agitation; and can contribute to elevations in systemic arterial and/or intracranial pressure [10]. These conditions also increase the risk of inadvertent self-extubation [78]. The use of ear protection and appropriate sedation may help mitigate these effects, alongside other flight-related stressors such as temperature fluctuations and vibration. Notably, low-frequency vibrations have been reported to potentiate the effects of thrombolytic therapy [24].

In addition, the aeronautical environment may heighten seizure susceptibility, primarily due to hypoxia, dehydration, pain, and agitation [4]. When seizure risk is present, air transport should be deferred until adequate stabilization has been achieved. Such patients should be transported while receiving appropriate antiepileptic therapy, with therapeutic serum drug concentrations confirmed before transfer [4,79]. Other neurological conditions that may deteriorate during flight include cerebral edema and cerebral vasospasm [80].

Isolation

The challenges encountered at 8,000 feet are quite different from those in hospital wards or ICUs [4,10]. In an aeronautical setting, patients are far from the equipment and support they might require, which can affect the quality of care due to these unique difficulties. Additionally, air transfers may require bypassing nearer facilities, resulting in longer travel distances to reach the nearest unit with adequate resources. Such delays could negatively affect patient outcomes [81]. Table 1 summarizes the aforementioned precautions and preventive measures into a practical survival guide.

**Table 1:** A practical survival guide for safe fixed-wing aeromedical transport of critically ill patients BP: Blood pressure; CPP: Cerebral perfusion pressure; CPB: Cardiopulmonary bypass; CT: Computed tomography; ETT: Endotracheal tube; HF: Heart failure; ICP: Intracranial pressure; LMWH: Low-molecular-weight heparin; NGT: Nasogastric tube; O₂: Oxygen; SCI: Spinal cord injury; SpO₂: Oxygen saturation; TBI: Traumatic brain injury.

System/Condition	Flight Stressor(s)	Adverse Events/Risks	Recommendations/Management
Respiratory/chronic lung disease, pneumothorax, recent thoracic surgery	Hypoxia, gas expansion	Desaturation if baseline SpO₂ < 92%; pneumothorax recurrence; expansion of trapped gas; respiratory compromise post-thoracic surgery	Continuous pulse oximetry; supplemental O₂ if SpO₂ < 92%; avoid smoking 48 h pre-flight; travel ≥2 weeks after pneumothorax resolution; travel ≥7–10 days after thoracic surgery; head/chest imaging before travel, if indicated
Cardiovascular-hematological/HF, thrombophilia, recent surgery, malignancy	Hypoxia, gravity forces, immobility	Dysrhythmias; syncopal episodes; venous/arterial thromboembolism; prothrombotic cascade activation	BP control; fluids; vasopressors (norepinephrine preferred in SCI); mobilization and leg exercises; hydration; compression stockings; LMWH for high-risk patients
Neurological/pneumocephalus, TBI, seizures, stroke, SCI	Hypoxia, gas expansion, gravity, noise/vibration, temperature	ICP and CPP fluctuations; neurological deterioration; seizures; agitation, anxiety; thermodysregulation	Delay travel ≥14 days after craniotomy or until pneumocephalus resolved; head CT before transfer; anticonvulsant therapy if seizure risk; sedation if intubated; earplugs; continuous monitoring; blankets; positioning
ENT - ophthalmology - dental/ear or sinus disease, ocular or dental procedures	Gas expansion (Boyle’s law)	Barotrauma: sinus pain, tympanic rupture, vertigo, hearing loss, dental pain, ocular complications	Delay travel ≥7–10 days after ENT or ocular surgery; avoid flight with acute infections; treat allergies or congestion before travel
Gastrointestinal - abdominal/recent surgery, ileus, obstruction	Gas expansion	Expansion of intestinal/gastric gas → suture disruption, perforation, ileus, barotrauma	Avoid early post-op flights (<7–10 days); NGT decompression if ileus/obstruction; hydration
Musculoskeletal - postsurgical/hip replacement, cesarean section, CPB surgery	Hypoxia, gas embolism, immobility	Decompression sickness; venous/arterial air embolism; wound dehiscence, infection	Delay flights until adequate wound healing, hydration, supplemental O₂, hyperbaric O₂ for decompression illness, if needed
Skin wounds	Hypoxia, gas expansion	Wound dehiscence; impaired healing, infection risk	Protect wounds; delay transfer if unstable or infected
Burns, infected wounds			
Renal - metabolic, electrolyte disorders, anemia	Hypoxia, dehydration	Worsening renal function; increased thromboembolic risk	Adequate hydration; monitor renal function; lab test before transfer; blood transfusion
General - environmental/critically ill patients	Temperature, humidity, noise, vibration, isolation	Dehydration; thickened secretions; difficulties with monitoring, communication, venous access; agitation; ICP increase	Cabin blankets; maintain cabin temp; humidification; fluids; proper positioning (head forward on takeoff, aft on landing); ensure backup equipment; automated or saline-filled ETT cuffs

## Conclusions

Clinicians are increasingly required to counsel patients on medical issues related to air travel, reflecting a steady rise in demand for such advice globally. This trend highlights the importance of familiarity with fundamental principles of aerospace physiology, driven by the expanding volume of commercial air travel and the growing proportion of elderly, disabled, and chronically ill passengers. While air transportation is regarded as highly safe relative to other modes of travel, appropriate patient counseling must take into account both flight-related environmental factors and individual health considerations.
